# Data concerning the effect of plyometric training on jump performance in soccer players: A meta-analysis

**DOI:** 10.1016/j.dib.2017.09.054

**Published:** 2017-09-30

**Authors:** Maamer Slimani, Armin Paravlić, Nicola Luigi Bragazzi

**Affiliations:** a Research Laboratory ‘‘Sport Performance Optimization’’, National Centre of Medicine and Science in Sport (CNMSS), El Menzah, Tunisia; b Science and Research Centre, Institute for Kinesiology Research, Garibaldijeva 1, 6000 Koper, Slovenia; c School of Public Health, Department of Health Sciences (DISSAL), Genoa University, Genoa, Italy; d Department of Neuroscience, Rehabilitation, Ophthalmology, Genetics, Maternal and Child Health (DINOGMI), Section of Psychiatry, Genoa University, Genoa, Italy

**Keywords:** Stretch-shortening cycle, Meta-analysis, Jump height, Soccer

## Abstract

Plyometric training (PT) enhances soccer performance, particularly vertical jump. However, the effectiveness of PT depends on various factors. A systematic search of the research literature was conducted for randomized controlled trials (RCTs) studying the effects of PT on countermovement jump (CMJ) height in soccer players. Ten studies were obtained through manual and electronic journal searches (up to April 2017). Significant differences were observed when compared: (1) PT group vs. control group (ES=0.85; 95% CI 0.47–1.23; *I*^2^=68.71%; *p*<0.001), (2) male vs. female soccer players (*Q*=4.52; *p*=0.033), (3) amateur vs. high-level players (*Q*=6.56; *p*=0.010), (4) single session volume (<120 jumps vs. ≥120 jumps; *Q*=6.12, *p*=0.013), (5) rest between repetitions (5 s vs. 10 s vs. 15 s vs. 30 s; *Q*=19.10, *p*<0.001), (6) rest between sets (30 s vs. 60 s vs. 90 s vs. 120 s vs. 240 s; *Q*=19.83, *p*=0.001) and (7) and overall training volume (low: <1600 jumps vs. high: ≥1600 jumps; *Q*=5.08, *p*=0.024). PT is an effective form of training to improve vertical jump performance (i.e., CMJ) in soccer players. The benefits of PT on CMJ performance are greater for interventions of longer rest interval between repetitions (30 s) and sets (240 s) with higher volume of more than 120 jumps per session and 1600 jumps in total. Gender and competitive level differences should be considered when planning PT programs in soccer players.

**Specifications Table**

**Table:** 

Subject area	*Sports sciences*
More specific subject area	*Sports physiology*
Type of data	*Raw and analyzed*
How data was acquired	*Data were acquired from articles included in the current meta-analysis.*
Data format	*Table*
Experimental factors	*Data concerning type of intervention, gender, age, and competitive levels, volume of training sessions based on number of jumps per single session, types of plyometric exercises, training program duration in weeks, weekly frequency of training, rest interval between repetitions, rest interval between sets, and overall training volume were extracted from the included studies.*
Experimental features	*Meta-analysis according to different moderator variables (type of intervention, gender, age, and competitive levels, volume of training sessions based on number of jumps per single session, types of plyometric exercises, training program duration in weeks, weekly frequency of training, rest interval between repetitions, rest interval between sets, and overall training volume) were performed.*
Data source location	*NA*
Data accessibility	*Data are within this article.*

**Value of the data**

• Largest plyometric training effects were verified in female and high-level soccer players compared to male and amateur counterparts, respectively.
• Higher volume of more than 120 jumps per session leads to greater effect of plyometric training on jump performance (countermovement jump (CMJ) ​without arm swing) when compared to less than 120 jumps per session.
• Longer rest interval between repetitions (30 s) and sets (240 s) provide larger improvements in jump performance than shorter rest.
• The benefits of plyometric training on jump performance are greater in participants who performed more than 1600 jumps in total than who performed less than 1600 jumps.

## 1 Data

Soccer is a most widely practiced sport around the world that combines cyclic and acyclic movements in competitive success [1], [2]. However, soccer players perform numerous explosive movements like kicking, tackling, jumping, turning, sprinting, and changing pace and directions during the match [2]. For instance, many athletic and technical movements in soccer require rapid rates of force production or power in addition to high levels of coordination and reactivity, which called plyometrics [3], [4].

When jump height performance was evaluated after plyometric training (PT), results from the literature were contradictory [3], [5]. For instance, the effectiveness of PT depends on various factors, such as age [6], competitive level [3], training volume [7], [8], and types of plyometric drills [3], [5], [9]. Therefore, the aim of this review was to examine the influence of various factors on the effectiveness of PT on jump height (i.e., countermovement jump (CMJ) without arm swing) using a meta-analysis approach.

## 2 Experimental design, materials and methods

### 2.1 Design, materials and methods

#### 2.1.1 Search strategy

**Fig. 1:**
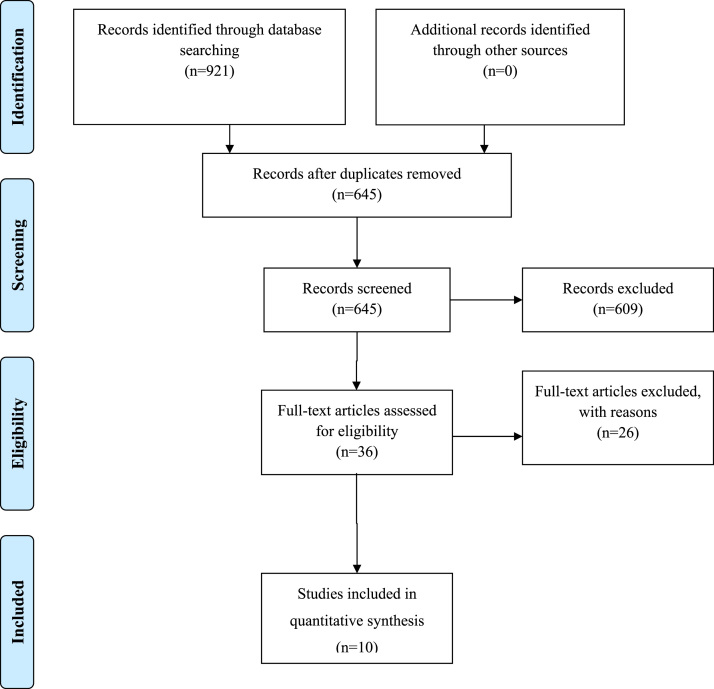
Preferred Reporting Items for Systematic Reviews and Meta-analysis (PRISMA) flow-chart.

The present meta-analysis was conducted according to the Preferred Reporting Items for Systematic Reviews and Meta-analysis (PRISMA) guidelines (Fig. 1, [10]). A systematic search of the research literature was conducted for randomized controlled trials (RCTs) studying the effects of PT on jump height in soccer players. Studies were obtained through manual and electronic journal searches (up to April 2017). The present review used the following databases: PubMed, SCOPUS, SportDiscus, PsycINFO, PsycARTICLES, Google Scholar, and ScienceDirect. Electronic databases were searched using keywords and/or MeSH terms: “plyometric” or “plyometrics” alone or together with “soccer”, “muscular power” and “jump”. Moreover, manual searches of relevant journals and reference lists obtained from articles were conducted. The present meta-analysis includes studies published in journals that have presented original research data on healthy human subjects. No age and gender were imposed during the search stage.

#### 2.1.2 Inclusion and exclusion criteria

Studies were included in the review if they met all the following Population/Intervention/Comparison/Outcome(s) (PICO) criteria:

(1) Population: studies recruiting male and female amateur (i.e., amateur, healthy, regional) and/or high-level (i.e., high-level, professional, national, 2^nd^ league, 1^st^ league) soccer players of any age as participants;
(2) Intervention or Exposure:
  (a) Investigations studying the effects of PT on CMJ height in soccer players;
  (b) Studies involving a control group against which an intervention could be compared;
(3) Comparison: CMJ height changes after PT according to the type of intervention, gender, age, and competitive level, volume of training sessions based on number of jumps per single session, types of plyometric exercises, training program duration in weeks, weekly frequency of training, rest interval between repetitions, rest interval between sets, and overall training volume;
(4) Outcome(s): CMJ height without arm swing after PT;
(5) Design: original investigations published in peer-reviewed journals;
(6) Language filter: English.

Studies were excluded if:

(1) Reviews, comments, opinions and commentaries, interviews, letter to editor, editorial, posters, conference abstracts, book chapters, and books were excluded; available reviews have been anyways scanned for increasing the chance of including potentially relevant articles;
(2) Assessing performance changed after PT combined with other intervention (strength or sprint training);
(3) Studies not employing true experimental designs and valid and reliable measurements;
(4) Studies including enough data to calculate effect size;
(5) Lacking quantitative information and details.

#### 2.1.3 Identification of the moderator variables

Two authors independently extracted data using a structured form. Because of the high number of variables that may affect training effectiveness, independent variables were grouped into the following areas: 1) type of intervention: plyometric group vs. control group; 2) subject characteristics: gender (male vs. female), age (<15 vs. between 15 and 21 vs. ≥21 years), and competitive levels (amateur vs. high-level); 3) program exercises: volume of training sessions based on number of jumps per single session (low: <120 vs. high: ≥120 jumps); types of plyometric exercises (single vs. combined exercises [two or more than two exercises]); and 3) program elements: a) training program duration in weeks (<8 vs. ≥8 weeks); b) weekly frequency of training (1 vs. 2 vs. 3 sessions per week); c) rest interval between repetitions (5 s vs. 10 s vs. 15 s vs. 30 s); d) rest interval between sets (30 s vs. 60 s vs. 90 s vs. 120 s vs. 240 s); e) overall training volume (low: <1600 jumps and high: ≥1600 jumps: calculated as total number of jumps per study).

#### 2.1.4 Statistical analyses

For the meta-analysis part, data were extracted from the included studies using a standardized documentation form. For preliminary analysis the effect estimates were computed as standardized mean differences of experimental and control group with their 95% confidence interval (CIs). Meta-analyses were carried out using the program Comprehensive Meta-Analysis, version 2 [11]. Statistical heterogeneity in our meta-analysis was assessed using the *Q* and *I*^2^ statistics. The *I*^2^ measure of inconsistency was used to examine between-study variability; values of 25, 50 and 75% represent low, moderate and high statistical heterogeneity, respectively [12]. Although the heterogeneity of effects in our meta-analysis ranged from 0% to 76.09% (see results section), we decided to apply a random-effects model of meta-analysis in all comparisons, in order to determine the pooled effect of PT on CMJ height. Possible publication bias was visually inspected with a funnel plot, looking at asymmetry of the graph. In addition, regression analysis (method of moments) were used in order to investigate possible predictors of observed effect size (ES) among aforementioned continuous variables (age of athletes, training program duration, weekly frequency, rest intervals between repetitions and sets, single session and overall training program volume. The magnitudes of the ESs were considered either trivial (<0.35), small (0.35–0.80), moderate (0.80–1.50), or large (>1.5) [13]. Furthermore, a regression analysis was used to verify the effects of potential moderator variables on the ES of study results. The significance level of *p*<0.05 was used.

The search strategies yielded a preliminary pool of 921 possible papers. The full text of 36 articles were retrieved and assessed for eligibility against the inclusion criteria. After a careful review of their full texts, 26 articles were excluded and the remaining 10 articles were eligible for inclusion in the review (Fig. 1; Table 1).

**Table 1:** Descriptive analysis of each plyometric study.

Study	Group	Gender	N	Age	Level	Type of jump	Weeks	Sessions per week	Number of jumps	Rest between rep (s)	Rest between sets (s)	CMJ (cm)	
												Pre	Post
Chelly et al. [14]	PG	M	12	19±0.7	H	C	8	2	40–100	5	NR	40±3	41±3
	CG	M	11	19±0.7	H		8					39±2	39±2
Fábrica et al. [15]	PG	NR	20	24.7±3.1	H	C	6	3	150–330	NR	NR	41.4± 2.5	44.4±1.7
	CG		20	24.7±3.1	H		6					41.3±2.0	41.5±2.1
Manouras et al. [16]	HPG	M	10	19.10±5.75	A	C	8	1	60–110	NR	60–120	30.7±3.00	31.7±2.9
	VPG	M	10	20.75±6.14	A	C	8	1	60–110	NR	60–120	29.2±7.10	30.9±6.7
	CG	M	10	20.00±3.5	A		8					32.1±6.80	32.5±6.8
Meylan and Malatesta [17]	PG	M	14	13.3±0.6	A	C	8	2	48–192	10	90	34.6±4.4	37.2±4.5
	CG	M	11	13.3±0.6	A		8					30.9±3.1	29.6±1.9
Negra et al. [18]	PG	M	11	12.8±0.3	A	C	4	2	112–280	10	90	22.89±6.06	24.35±5.02
	CG	M	11	12.7±0.3	A		4					21.13±2.96	22.01±3.59
	PG	M	11	12.8±0.3	A	C	8	2	112–280	10	90	22.89±6.06	26.57±5.56
	CG	M	11	12.7±0.3	A		8					21.13±2.96	23.75±3.34
	PG	M	11	12.8±0.3	A	C	12	2	112–280	10	90	22.89±6.06	28.17±5.93
	CG	M	11	12.7±0.3	A		12					21.13±2.96	21.99±1.88
Ozbar [19]	PG	F	10	19.3±1.6	H	C	10	2	120–250	NR	NR	40.1±1.9	48.6±1.6
	CG	F	10	19.3±1.6	H		10					39.7±1.8	42.3±1.9
Ozbar et al. [20]	PG	F	9	15–22	H	C	8	1	90–220	NR	NR	39.8±4.5	46.8±2.2
	CG	F	9	15–22	H		8					35.4±4.6	37.9±3.9
Ramirez-Campillo et al. [21]	PG 30	M	13	10.4±2.0	A	S	7	2	60	15	30	22.2±4.1	24.0±5.6
	PG 60	M	13	10.4±2.3	A	S	7	2	60	15	60	21.9±2.1	23.9±3.1
	PG 120	M	11	10.3±2.3	A	S	7	2	60	15	120	21.7±4.4	23.5±5.4
	CG	M	14	10.1±2.0	A		7					22.1±4.9	21.9±4.7
Ramírez-Campillo et al. [22]	FCG	F	19	20.5±2.5	A		6			15	60	26.6±4.8	26.6±4.3
	FPG	F	19	22.4±2.4	A	C	6	2	80–120	15	60	26.7±5.5	29.4±5.8
	MCG	M	21	20.8±2.7	A		6			15	60	33.2±3.9	32.8±3.8
	MPG	M	21	20.4±2.8	A	C	6	2	80–120	15	60	35.3±3.3	37.6±4.0
Sedano Campo et al. [23]	PG	F	10	23.0±3.2	H	C	6	3	200–330	30	240	25.6±1.0	27.8±0.9
	CG	F	10	22.8±2.1	H		6					26.2±0.9	24.7±1.0

A: amateur; C: combined jumps; CMJ: countermovement jump; F: female; FCG: female control group; FPG: female plyometric group; H: high-level; HPG: horizontal plyometric training group; M: male; MCG: male control group; MPG: male plyometric group; NR: not reported; rep: repetitions; S: single jump; Time: seconds(s); VPG: vertical plyometric training group.

### 2.2 Type of intervention (plyometric training group vs. control group)

**Fig. 2:**
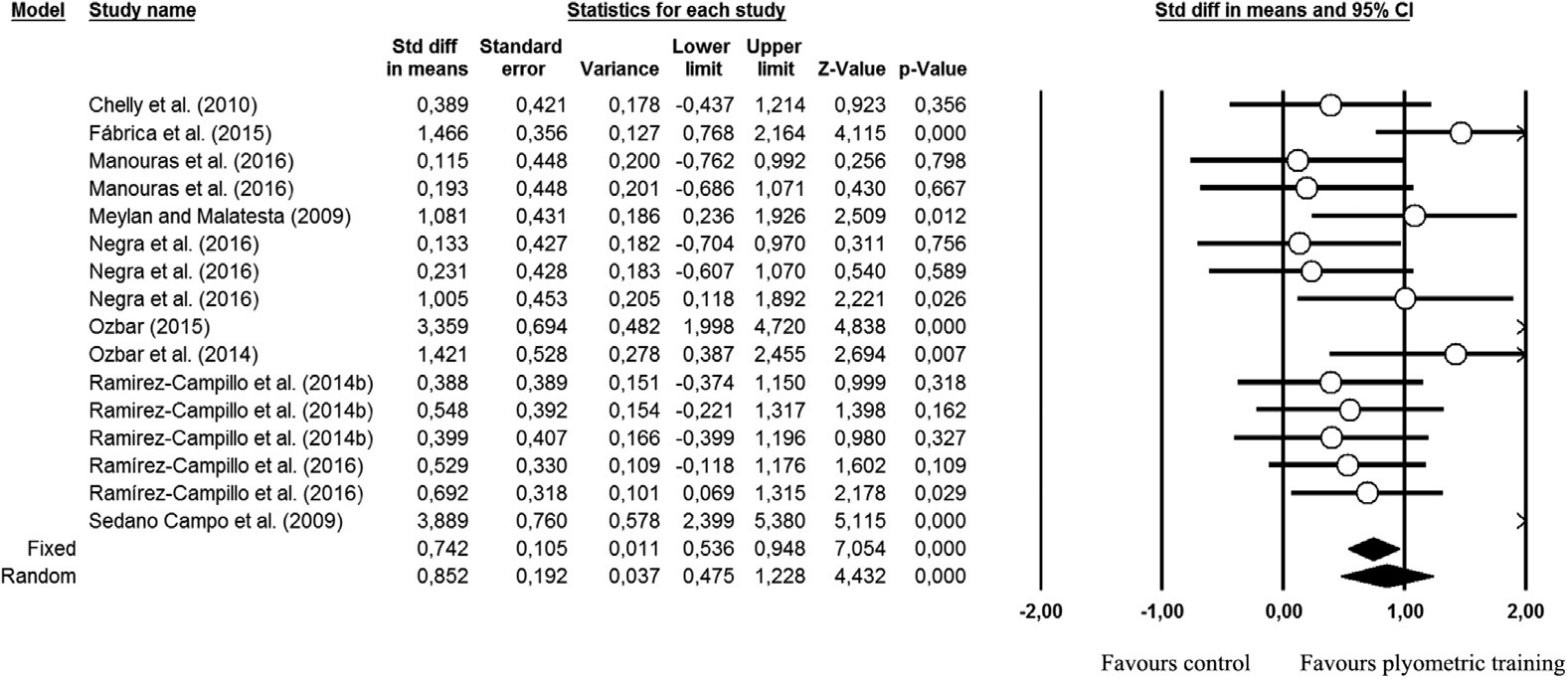
Effects of plyometric training *vs.* control group on maximal CMJ height. Std: Standard; diff: difference; CI: confidence interval.

The meta-analyzed effect of PT was moderate on CMJ height (ES=0.85; 95% CI 0.47–1.23; *I*^2^=68.71%; *p*<0.001), when compared to control group (Fig. 2).

### 2.3 Gender (male vs. female)

Greater effect of PT was observed for females compared to male soccer players (*Q*=4.52; *p*=0.033) (Table 2).

**Table 2:** Effects of plyometric training considering different grouping variables.

Independent variables	ES	SD	95% CI	*p*	*I*^*2*^ (%)	*df*	*Q value and (p) between groups*
**Gender**							**4.52 (0.033)**
Female	2.20	1.59	0.64 to 3.77	0.006	88.30	3	
Male	0.48	0.33	0.03 to 1.68	<0.001	0.00	10	
**Age of athletes**							
<15 years	0.53	0.27	0.22 to 0.84	0.001	0.00	6	
15–21	1.23	1.47	0.01 to 2.45	0.049	83.39	3	
≥21	1.17	1.49	0.34 to 2.01	0.006	81.94	4	2.98 (0.225)
**Competitive levels of athlete**							
Amateur	0.50	0.33	0.26 to 0.73	<0.001	0	10	**6.56 (0.010)**
High level	1.98	1.46	0.87 to 3.09	0.025	83.31	4	
**Type of exercise**							
Single	0.98	1.21	0.51 to 1.44	<0.001	73.74	12	
Combined	0.45	0.09	0.00 to 0.89	0.051	0.00	2	2.58 (0.108)
**Single session volume**							
<120 jumps	0.45	0.19	0.18 to 0.71	0.001	72.57	7	
≥120 jumps	1.44	1.36	0.70 to 1.03	<0.001	68.77	7	**6.12 (0.013)**
**Training program duration**							
<8 weeks	0.84	1.23	0.32 to 1.37	0.002	72.57	7	
≥8 weeks	0.87	1.08	0.29 to 1.46	0.003	68.77	7	0.00 (0.945)
**Weekly frequency of training**							
1 per week	0.53	0.73	−0.25 to 1.31	0.181	53.04	2	**2.62 (0.270)**
2 per week	2.00	0.90	0.32 to 1.02	<0.001	51.62	10	
3 per week	3.00	1.71	0.22 to 4.95	0.032	88.00	1	
**Rest between repetitions**							
5 s	0.39	a	−0.44 to 1.21	0.356	0.00	0	**19.10 (<0.001)**
10 s	0.60	0.50	0.11to 1.09	0.016	24.76	3	
15 s	0.53	0.12	0.21to 0.85	0.001	0.00	4	
30 s	3.89	a	2.40to 5.38	<0.001	0.00	0	
**Rest between sets**							
30 s	0.39	a	-0.37to 1.15	0.318	0.00	0	
60 s	0.60	0.09	0.21to 0.98	0.003	6.55	2	
90 s	0.46	0.45	0.09to 0.82	0.014	0.00	5	
120 s	0.40	a	-0.40 to 1.20	0.327	0.00	0	
240 s	3.89	a	2.40 to 5.38	<0.001	0.00	0	**19.83 (0.001)**
**Overall training program volume**							
Low <1600 jumps	0.52	0.40	0.28 to 0.76	<0.001	0.00	10	
High ≥1600 jumps	1.55	1.57	0.72 to 3.00	0.001	85.14	4	**5.08 (0.024)**

*CI* confidence interval, *ES* effect size, *I*^*2*^ index of heterogeneity, *N* number, *P* significance level, *SD* standard deviation

a No variance, because only one ES was included in analysis.

### 2.4 Age of athletes

No significant difference was observed between age groups (<15 vs. 15–21 vs. ≥21 years; *Q*=2.98, *p*=0.225) in CMJ height after PT.

### 2.5 Competitive level (amateur vs. high-level)

Significant difference was found between high-level soccer players compared to amateur counterparts (*Q*=6.56; *p*=0.010) in CMJ height after PT.

### 2.6 Types of plyometric exercises (single vs. combined exercises)

There was no significant difference in magnitude of ES when studies with single or combined exercises (*Q*=2.58; *p*=0.108) were compared.

### 2.7 Single training session volume

**Fig. 3:**
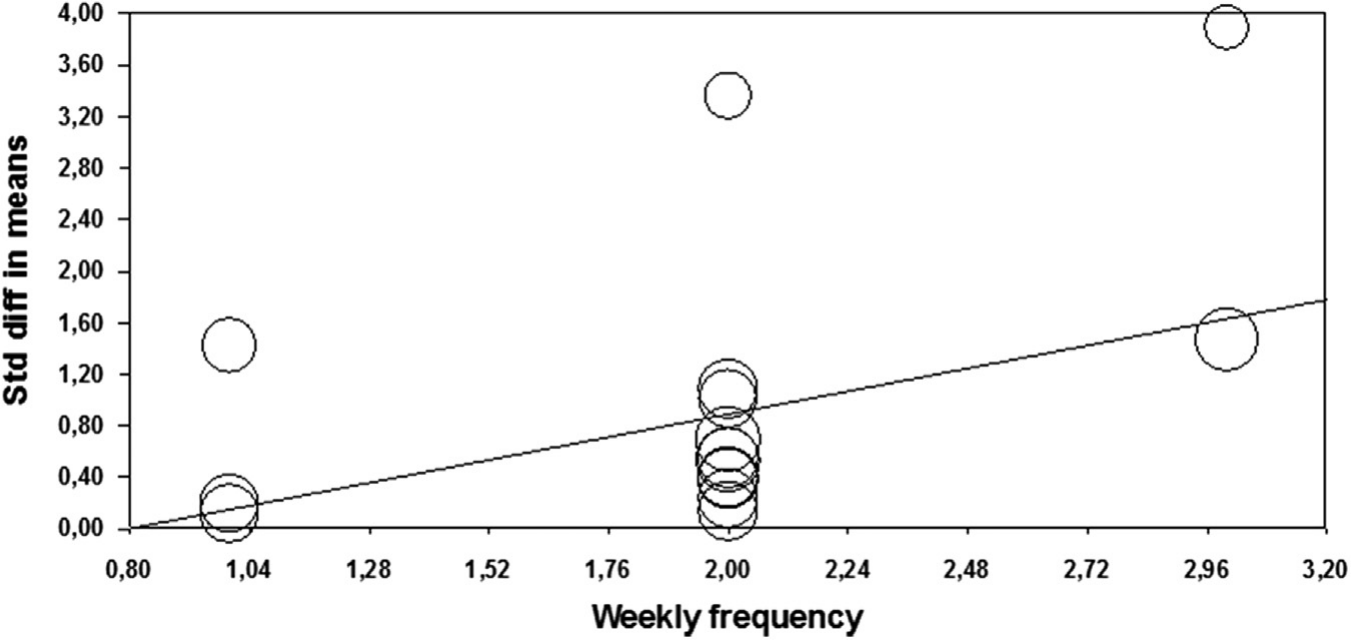
Meta-regression performed with weekly frequency as moderator. Std: Standard; diff: difference.

There was significant effect of number of jumps per training session, where higher volume of more than 120 jumps leads to greater effect when compared to less than 120 jumps per training session (*Q*=6.12; *p*=0.013) (Table 2). In addition, regression analysis confirmed preliminary results, showing that number of jumps as significant predictor of the ES (*Z*=2.998; *p*=0.003) (Table 3; Fig. 3).

**Table 3:** Meta regression for training variables of different subscales to predict plyometric training effects on CMJ height.

	Beta Coefficient	Standard error	95% lower CI	95% upper CI	*Z* value	*P* value
Age of athletes	0.0670	0.039	−0.010	0.144	1.696	0.090
Training programme duration	0.0899	0.113	−0.132	0.311	0.795	0.427
Weekly frequency of training	0.7402	0.337	0.079	1.401	2.194	**0.028**
Rest interval between reps	0.0992	0.034	0.032	0.167	2.885	**0.004**
Rest interval between sets	0.0124	0.004	0.005	0.020	3.079	**0.002**
Single session volume	0.0078	0.003	0.003	0.013	2.998	**0.003**
Overall training programme volume	0.0004	0.000	0.000	0.001	3.339	**<0.001**

### 2.8 Program duration (<8 vs. ≥ 8 weeks)

No significant difference was observed between <8 vs. ≥ 8 weeks duration comparison (*Q*=0.00; *p*=0.945).

### 2.9 Frequency of weekly sessions (1 vs. 2 vs. 3 sessions per week)

**Fig. 4:**
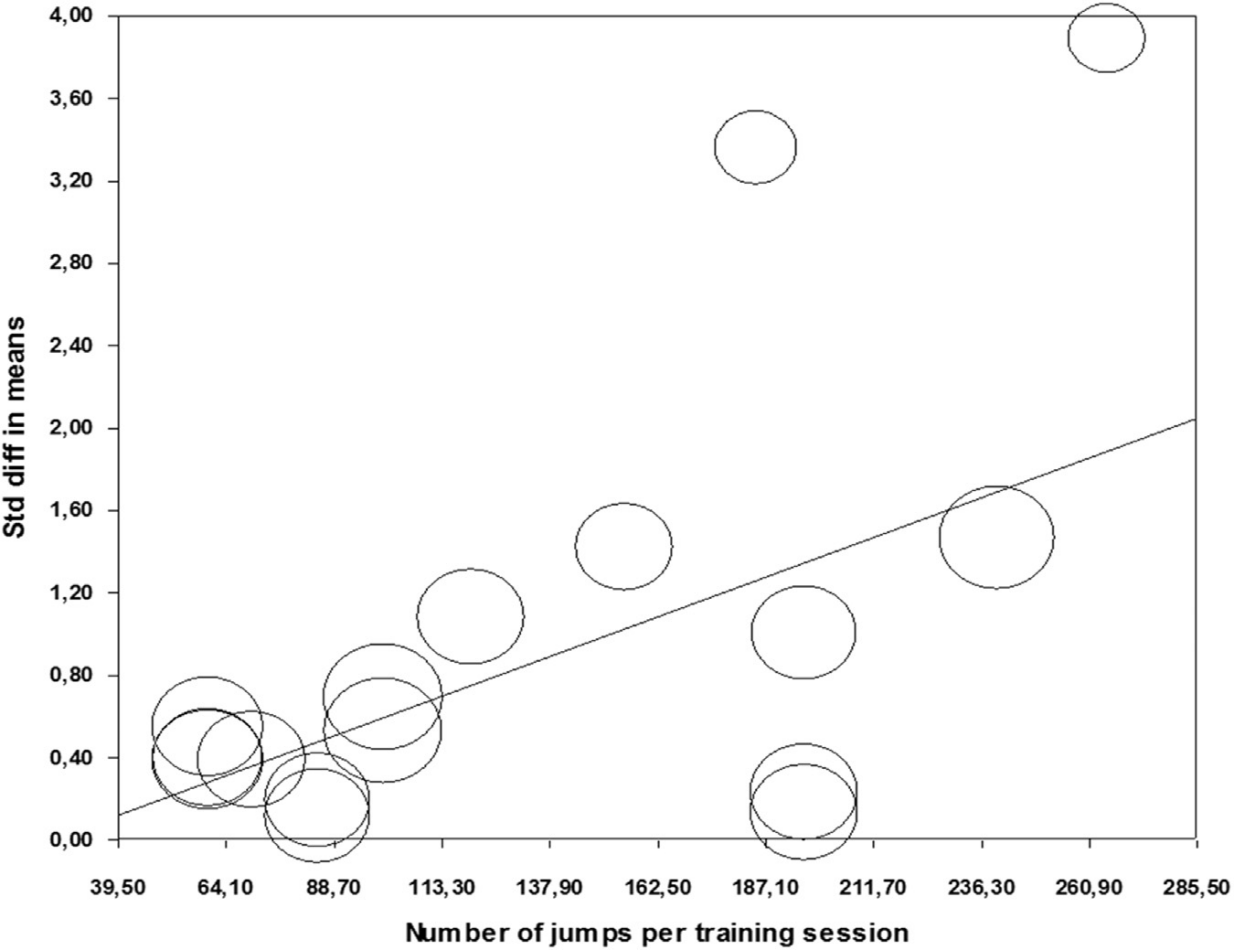
Meta-regression performed with single session volume as moderator. Std: Standard; diff: difference.

Weekly frequency of training showed heterogeneous effects ranging from small (ES=0.53) to large (ES=3.00) for one to three trainings per week, respectively, without significant difference between them (*Q*=2.62; *p*=0.270). However, regression analysis showed that weekly frequency of training was significant predictor (*Z*=2.194; *p*=0.028) of CMJ height gains following PT (Fig. 4).

### 2.10 Rest between repetitions (5 s vs. 10 s vs. 15 s vs. 30 s)

**Fig. 5:**
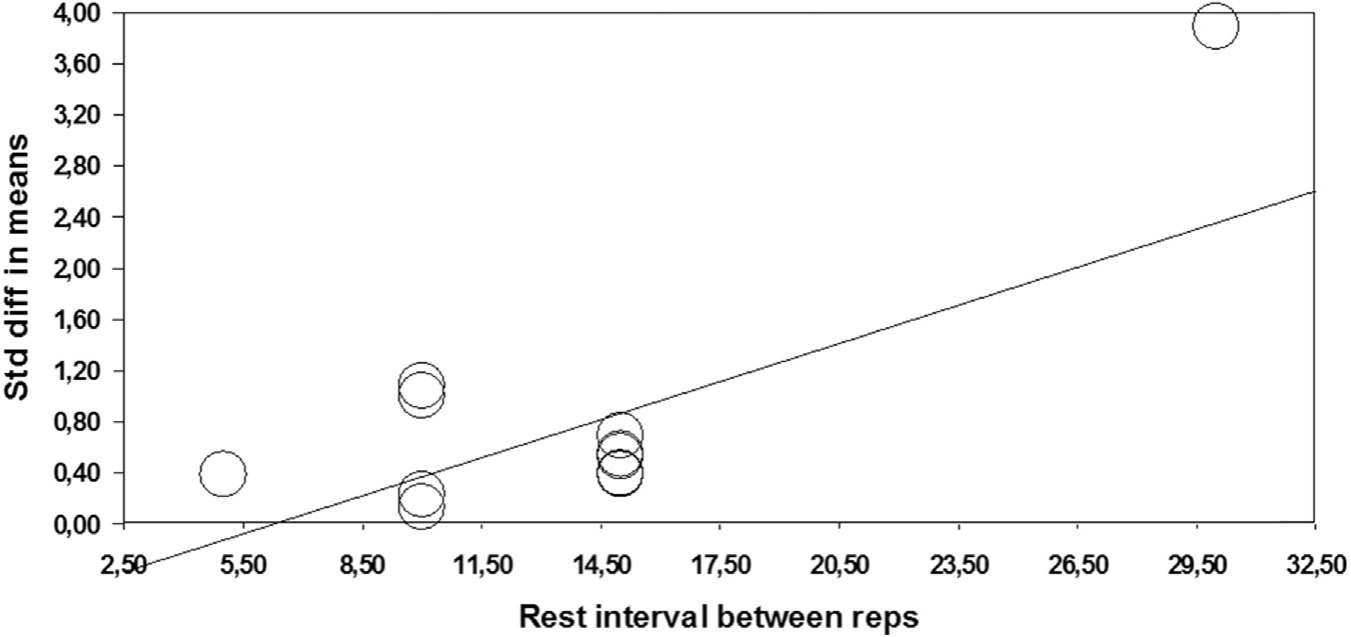
Meta-regression performed with rest interval between repetitions (reps) as moderator. Std: Standard; diff: difference.

Meta-analyzed effect of PT regarding different rest periods between repetitions yielded heterogeneous effects ranging from small (ES=0.39; 5 s) to large (ES=1.06; 30 s) (Table 2). Compared to all other intervals, 30 s of rest between repetitions showed significant difference in gains (*p*<0.001, Q ranging from 16.85 to 18.68 for 10 s and 15 s intervals). In addition, regression analysis have shown that rest interval between repetitions is significant predictor of observed effect (*Z*=2.885; *p*=0.004) (Table 3; Fig. 5).

### 2.11 Rest between sets (30 s vs. 60 s vs. 90 s vs. 120 s vs. 240 s)

**Fig. 6:**
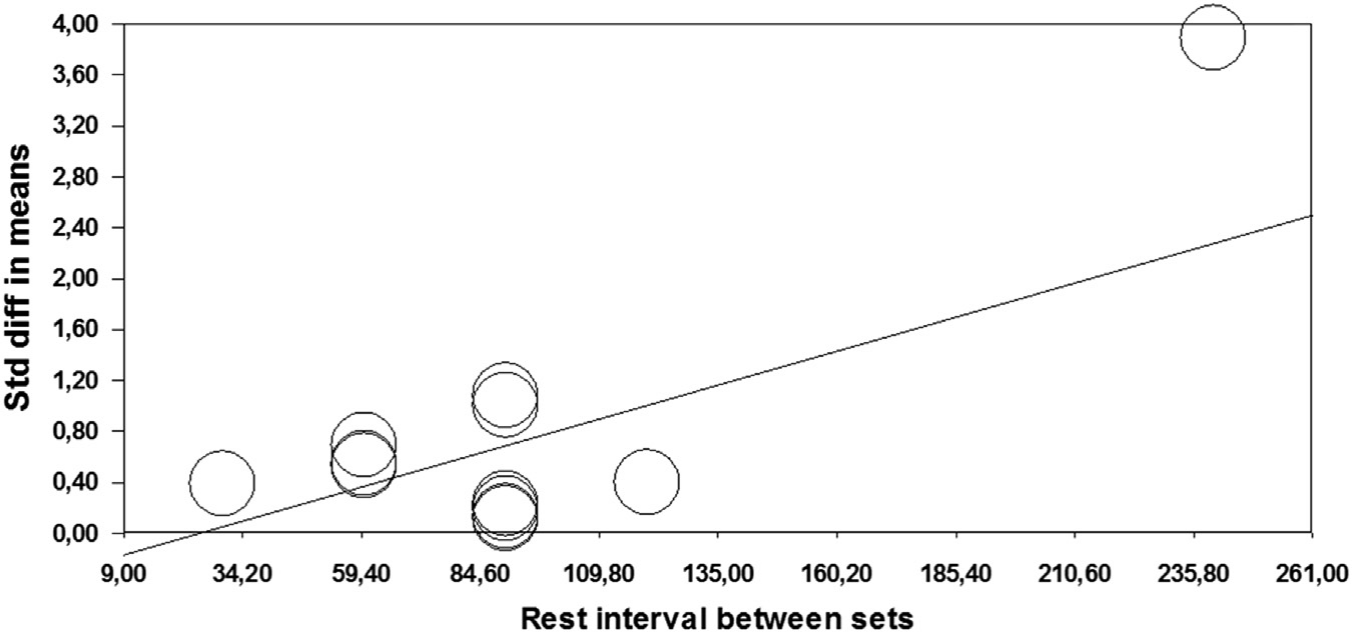
Meta-regression performed with rest interval between sets as moderator. Std: Standard; diff: difference.

The rest between sets showed diversity of effect ranging from small (ES=0.39, 30 s) to large (ES=3.89; 240 s). Compared to all other intervals significant difference was observed only for rest of 240 s between sets (*p*<0.001, *Q* ranging from 16.384 to 19.233). In addition, regression analysis have shown that rest interval between sets is significant predictor of observed effect (*Z*=3,079; *p*=0.002) (Table 3; Fig. 6).

### 2.12 Overall training program volume

**Fig. 7:**
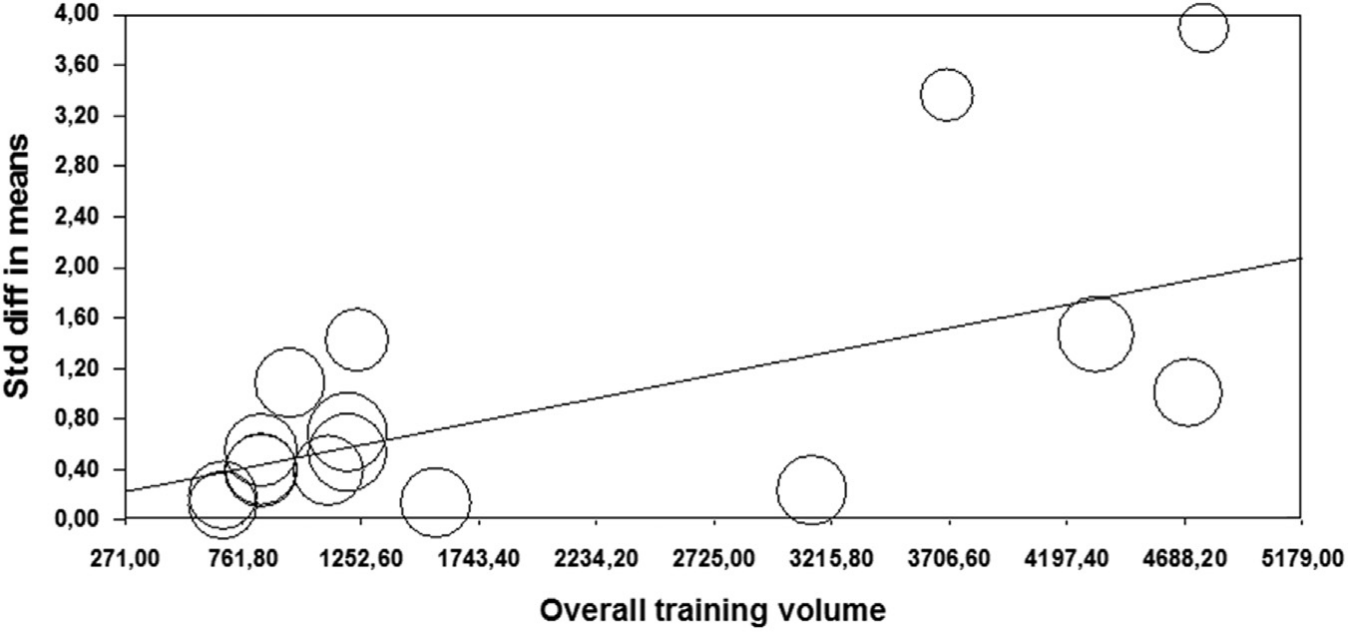
Meta-regression performed with overall training program volume as moderator. Std: Standard; diff: difference.

Marginally significant effect of training volume was observed among analyzed studies, where more than 1600 jumps showed greater ES (ES=1.55) compared to less than 1600 jumps per study (ES=0.52). Additional regression analysis indicated overall training volume as significant predictor of observed effects (*Z*=3.339; *p*=0.001) (Table 3; Fig. 7).

Despite the important conclusions that can be drawn from this meta-analysis concerning the effectiveness of PT on jump performance, it is important to note that some limitations should be considered. For example, there was a considerable amount of small numbers of included studies, and this can be due to the high-quality standards used to select studies (e.g., randomized-controlled), and the highly specific focus of the data (i.e., CMJ, in soccer). This leads to bias or limitations in the generalization of results.
